# A qualitative study of perspectives on access to tuberculosis health services in Xigaze, China

**DOI:** 10.1186/s40249-021-00906-4

**Published:** 2021-09-20

**Authors:** Victoria Haldane, Zhitong Zhang, Qi Ma, Tingting Yin, Bei Zhang, Yinlong Li, Qiuyu Pan, Katie N. Dainty, Elizabeth Rea, Pande Pasang, Xiaolin Wei, Jun Hu

**Affiliations:** 1 Institute of Health Policy, Management and Evaluation, University of Toronto, 155 College St., Toronto, ON M5T 3M6 Canada; 2 Dalla Lana School of Public Health, University of Toronto, 155 College St., Toronto, ON M5T 3M7 Canada; 3 Weifang Medical College, Weifang, Shandong China; 4 Jining Medical University, Jining, Shandong China; 5 North Sichuan Medical College, Nanchong, Sichuan China; 6 Xigaze Centre for Disease Control and Prevention, 7 Keji Road, Sangzhuzi District, Xigaze, Xizang China; 7 Shandong University of Traditional Chinese Medicine, Jinan, 250355 China

**Keywords:** Tuberculosis, Access, Quality of care, Qualitative research, Rural health, China

## Abstract

**Background:**

Tuberculosis (TB) is a major global health threat and the leading infectious disease cause of death worldwide. Access to and retention in TB care remains a challenge for patients, particularly those living in rural and remote settings. This qualitative study explored barriers and facilitators to accessing and maintaining contact with TB care services in communities in Xigaze (Shigatse) prefecture, Xizang Autonomous Region (Tibet Autonomous Region), China from the perspective of persons impacted by TB.

**Methods:**

We conduced in-depth interviews with 23 participants impacted by TB in four rural districts in Xigaze prefecture, Xizang Autonomous Region, China between April 2019 and November 2020. Interviews were conducted in Tibetan and Mandarin, transcribed in Mandarin and translated into English. Transcripts were checked against recordings by native Tibetan and Mandarin speakers. QSR NVivo12 software was used for framework analysis guided by an access to care conceptual framework by Levesque et al.

**Results:**

Overall patients reported low awareness of and an indifferent attitude towards TB, although all reported understanding the need to adhere to treatment. Participants reported complex pathways to care, often requiring visits to multiple healthcare facilities. Some participants reported visiting traditional Tibetan medicine (TTM) providers. Participants reported various barriers to accessing care including challenges physically reaching care, out-of-pocket payments for tests, diagnostics and transport. Barriers to maintaining care included medication side effects and worry about treatment effectiveness. Enablers to accessing care identified included knowledge or past experience with TB, integrated models of TTM and western care, supportive village doctors who conducted home visits, free TB treatment and other subsidies, as well as having family support with care and social support as barriers and facilitators to maintaining treatment.

**Conclusions:**

We identified barriers and facilitators to accessing services in rural communities in Xigaze from the perspective of persons impacted by TB. Challenges include complex pathways to care, travel distances, wait times and low awareness. Tuberculosis care in the region could be strengthened by ongoing culturally tailored educational campaigns to increase awareness, partnerships with TTM providers, providing comprehensive treatment subsidies and strengthening the role of family members in comprehensive TB care.

**Graphic abstract:**

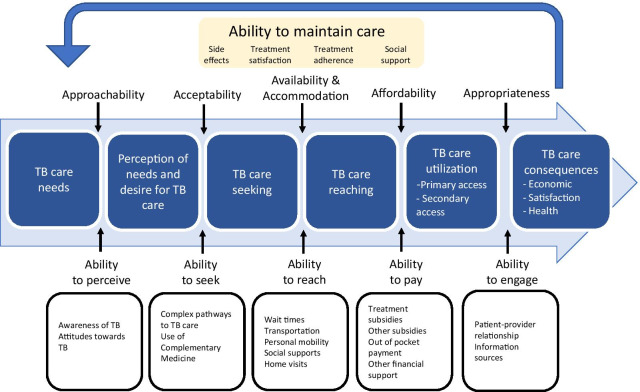

**Supplementary Information:**

The online version contains supplementary material available at 10.1186/s40249-021-00906-4.

## Background

Tuberculosis (TB) remains a major threat to global health and well-being [1]. Effective TB control programs require early diagnosis and prompt treatment initiation. However, in 2018 it was estimated that three million TB cases were un-diagnosed, mainly in low-and-middle income countries (LMICs), a figure that has likely increased due to the impact of COVID-19 on care seeking and TB diagnosis [2, 3]. Diagnostic delay, or delay in the time between symptom onset and treatment, can lead to greater clinical severity when presenting for treatment and have impacts on treatment outcomes [4]. A 2017 review of LMICs found that 42% of pulmonary TB patients delayed seeking care by a month or more, and that the median diagnostic delay ranged from 30 to 366.5 days (IQR 44–77.8) [5]. This delay may be patient-related, resulting from barriers to accessing healthcare or stigma [6]. Or, when patients are able to access care, delays may be the result of healthcare and health systems related barriers including poor TB screening guidelines, inadequate testing infrastructure, or care organization factors [7, 8]. These may also influence pre-treatment loss to follow up in LMICs, with 2014 estimates reporting a range of 4% to 38% of cases lost to follow up before initiating treatment [9].

Once treatment has been initiated, adherence to medication regimes is crucial to achieving favourable patient outcomes, reducing drug resistance and ending the global TB epidemic [10]. However, medication adherence often proves challenging for patients and their families for a variety of reasons, including lengthy regimes, treatment side effects, lack of treatment support, stigma and privacy concerns [11, 12]. Directly observed treatment (DOT), in which patients are observed taking their medications by a health worker or other treatment supporter, is the cornerstone of adherence support for TB programs worldwide. However, conventional DOT presents challenges as it requires regular and frequent in-person follow-up over six months or longer. This lengthy follow-up can be resource intensive and present challenges for patients, their families and the health systems that offer care, particularly in resource-constrained settings [13–15]. Indeed loss to follow up is a crucial challenge, and it has been estimated 26% of all cases are lost to follow up in the World Health Organization (WHO) Western Pacific region, whereas other regions reported between 4 and 6% loss to follow up [1].

Taken together, there are significant challenges to both patient access to and maintenance of TB care across the care cascade [16]. A range of interventions, including those using peer support or digital technologies, have been employed to improve the quality of TB care delivered. These interventions include programs to support persons with TB in adhering to their medications, create more person-centred TB services and bridge gaps in TB care worldwide, particularly in LMICs [17]. The importance of social and cultural factors in TB control and successful TB programme implementation has long been known [18]. Community involvement in research and programme development, in particular by those most impacted by TB, has become increasingly normalized as a way to ensure equitable and culturally appropriate access to care [19]. However, there continues to be a need for a more complete understanding of the feasibility of programs through the lens of the contexts in which they are implemented and the lived realities of the people whom these programs serve, particularly those living in areas with limited access to care and a high burden of TB [20].

The Xizang Autonomous Region (Xizang), also known as the Tibet Autonomous Region, China is located on the Tibetan plateau and is a region characterized by its remoteness, high altitude, and low population density. The health system in Xizang has made significant gains in population health in recent years, with falling rates of maternal and infant mortality, and life expectancy rising from 35.5 years in the 1960s to 67 years in 2014 [21]. However, TB remains a major challenge to health and well-being in Xizang. The most recent TB prevalence study in Xizang, conducted in 2014, showed that the pulmonary TB prevalence rate was 758/100 000 population, almost twice China’s national average (442/100 000), and orders of magnitude larger than low-burden countries such as Canada (4.6/100 000) [22–24].

This qualitative study aims to provide experiential evidence of factors influencing the ability of persons with TB to access and maintain their care in rural Xigaze (Shigatse) prefecture, Xizang, China as part of a broader intervention to improve the quality of TB care in the region.

## Methods

### Setting

Xigaze is a prefecture in Xizang. It is located to the west of Lhasa and borders Nepal and the Himalaya mountain range, including Mount Everest. The population of approximately 800 000 are spread out over an area of 182 000 km^2^, which translates to a low population density of roughly 4 persons/km^2^ in the prefecture. In Xigaze, all patients are treated as per China National Tuberculosis Program (NTP) guidelines. The NTP provides free anti-TB medicines and a defined number of sputum tests and chest X-rays for TB patients. Patients need to pay for other medicines and extra tests, which are partly covered by their medical insurance. In mid-2019 the organization of TB care in Xigaze, and Xizang overall, shifted away from diagnosis at local Centres for Disease Control (CDC) and now county hospitals assume responsibility for TB diagnosis. Once diagnosed at the county hospital, patients are referred to their township hospital where a township doctor oversees their ongoing TB treatment. The township doctor will notify the patients’ village doctor, who then follows up with the patient at their home. Village doctors primarily provide the patient with support, monitor whether they are having challenges adhering to their medications, and provide basic management of side effects.

However, in practice, TB care in the region has proven difficult to operationalize for many reasons. Challenges impacting Xizang more broadly, and Xigaze specifically, include a shortage of skilled health workers to provide comprehensive ongoing care, inadequate diagnostic capacity and lab facilities, as well as harsh terrain and weather conditions, which disrupt patient and health workers’ travel thereby limiting ongoing care [25, 26]. Despite NTP guidelines calling for DOTS and monthly follow-up, most patients receive self-administered therapy with limited health system contact, which has contributed to a high loss to follow-up across the continuum of TB care. Recent data from Xigaze reported that, in 2016, only 72% (769/1073) of new pulmonary TB cases completed treatment. Of those who did not complete treatment the majority, 83% (252/304), were lost to follow-up [25]. Thus, there are significant gaps in TB control and service delivery across Xigaze. Further, there is limited evidence on the patient perspective of TB care in Xigaze, with previous studies focusing on factors relating to non-adherence in Xizang generally [26].

#### Conceptual framework

**Fig. 1:**
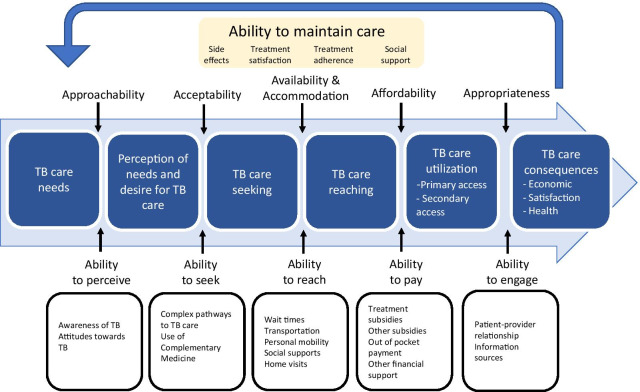
Re-conceptualization of Levesque et al. for ability to access tuberculosis (TB) care.

This study employs a framework by Levesque et al. to conceptualize patients’ ability to access to care, which has previously been used in other studies of access to TB treatment amongst rural populations [27, 28]. The framework presents five abilities that define accessibility. These abilities represent an individuals’ ability to interact with the dimensions of accessibility, which together represent access to care. As this study relies on patient-reported perspectives and aims to understand dimensions of their abilities to access care, we use the patient-oriented ‘demand’-side constructs of the framework with sub-themes identified from our analysis. We expand upon this framework by including one inductively developed theme to characterize the way in which the consequences of TB care may support or limit patient abilities to maintain their treatment in our setting (Fig. 1).

We frame ability to perceive the need for care as participants’ awareness of and attitude towards TB. Awareness of TB and attitude towards having TB interact to influence whether a person with symptoms perceives care is needed, and once diagnosed, whether ongoing care is necessary. Ability to seek refers to knowledge of available healthcare options. These options are shaped by the health care system, local culture, as well as societal norms and values [27]. In China, people are able to directly access tertiary care without referral and as such, our participants sought care from a variety of sources when presenting with symptoms. Ability to reach refers to how once a person perceives that they require care for their symptoms, there are multiple factors that may enable or deter them from physically reaching the care they would like to access [27]. Ability to pay refers to a persons’ capacity to generate economic resources, be it through income, formal or informal borrowing, to pay for health services without exposure to financial hardship or opportunity costs related to loss of income while accessing care [27]. Once patients access care, their ability to engage with their care is directly informed by their interaction with their health care provider and their sources of health-related information. Ability to maintain care is an inductively described theme which highlights how consequences of care such as side effects, satisfaction with treatment and adherence interact to enable patients’ ability to continue and maintain access to care over a complete course of treatment. This ability is also facilitated by social support.

#### Selection of participants, data collection and processing

This study was conducted between April 2019 and November 2020. Participants were selected amongst those older than 15 years old, with presumed TB and newly confirmed pulmonary TB starting on standard 6-month short-course outpatient treatment. Recruitment aimed for a balance of gender, age, and location across four counties representing a mix of urban and rural locations. Participants were invited to the study by their township hospital doctors through phone call. The study team made efforts to ensure interviews took place in comfortable, private locations suitable to the respondent. Data was securely stored as per protocols defined by the University of Toronto Office of Research Ethics including password protection and use of secure drives.

Interviews were conducted by trained researchers from the study team using a pre-designed interview guide covering access to care related constructs (Box 1). After obtaining informed consent, the interviews were audio recorded. The interviews were conducted by research staff in Mandarin with interpretation to Tibetan at the time of interview. All interviews were recorded and transcribed in full into Mandarin by the interviewers and then translated into English by research staff. Transcripts were checked against the original recordings by a bilingual translator to ensure accuracy between recordings and transcriptions.

**Table:** 

Box 1: Summary interview guide	
1. Can you tell me about how you were diagnosed with TB?	
2. Where were you diagnosed?	
3. How often do you see your doctor?	
4. What is your opinion of your doctor?	
Follow up	
Do you feel they give you appropriate advice? Why/not?	
Do you feel like they understand your concerns and respond to your questions?	
Do you feel like they understand your concerns? Why/not?	
5. Do you face any difficulties going to see the doctor?	
6. How do you pay for your medical expenses?	
Follow up	
Does anyone help you pay for your medical expenses? Who?	
Do you feel your medical expenses are affordable? Why/Why not?	
7. How much in total have you spent on medical expenses?	
8. Do you have any other expenses related to your health?	
9. Has anything about your lifestyle changed since you were diagnosed with TB?	
10. Could you share with us all the medicine you currently take?	
11. What instructions were you given on how to take the medication?	
12. Do you experience any problems in taking the medication?	
13. Do you ever forget to take your medicine?	
14. Does anyone or anything remind you to take your medication?	
15. Do you take any traditional medicines?	
16. Overall, are you satisfied with your TB treatment?	
Additional question added for interviews conducted after January 2020	
17. How has COVID-19 impacted your TB treatment?	

The analysis adopted a qualitative descriptive approach, which has previously been used in conducting cross-cultural qualitative health services research [29]. Two research team members (VH, QM) coded interviews deductively using framework analysis as described by Ritchie and Lewis, while allowing for elements of thematic analysis as described by Braun and Clarke, namely inductive identification of themes and sub-themes [30, 31]. Data was organized and coding conducted using the qualitative research software NVivo 12 (QSR International, Doncaster, Australia). We used the abilities components of Levesque’s access to care framework to guide our coding framework and employed an iterative process to determine any other relevant codes. Two reviewers (VH, QM) independently coded the first three transcripts using the coding framework and then discussed any inductive additions. After discussion and agreement, the modified coding framework was applied to subsequent manuscripts. We stopped interviewing when it was decided that no new concepts were being heard in subsequent interviews [32].

## Results

A total of 23 interviews were conducted with persons impacted by TB in Xigaze including patients and family members who act as treatment supporters. The study was conducted in three rural counties (Gyangze, Sa’gya, and Tingri) and one urban district (Sangzhuzi). Interviewees were purposively sampled based on district (Table 1).

**Table 1:** Description of qualitative interview participant characteristics

Participant characteristics	Female	Male	Total
Sex	11	12	23
Age, years			
Unknown	2	1	3
0–30	3	1	4
30–40	0	1	1
40–50	1	4	5
50–60	2	4	6
60 +	2	1	3
Location			
Sangzhuzi (Urban)	1	6	7
Sa’gya (Rural)	2	3	5
Gyangze (Rural)	3	1	4
Tingri (Rural)	5	2	7
Participant type			
Patient	6	12	18
Family treatment supporter	4	1	5
Relationship to patient			
Spouse	1	0	1
Child/Child-in-law	1	0	1
Sibling	1	1	2
Aunt/Uncle	1	0	1

We organized our data using six main themes and twenty sub-themes as per our adapted conceptual framework. Table 2 provides an overview of our themes and the sub-themes explored therein.

**Table 2:** Overview of themes used in the analysis

Theme	Sub-themes
Ability to perceive	• Awareness of tuberculosis • Attitudes towards tuberculosis
Ability to seek	• Healthcare options • Pathways to care
Ability to reach	• Physical mobility related to their health condition • Transport options • Wait times • Family support • Home visits by village doctors
Ability to pay	• Government subsidies • Out of pocket payments • Transport costs • Opportunity costs in seeking care
Ability to engage	• Doctor-patient relationship • Lived experiences • Information sources
Ability to maintain care	• Side effects • Satisfaction with treatment • Social support

### Ability to perceive: attitude towards TB

Participants reported various symptoms which prompted them to seek care. Most patients reported vague symptoms such as ‘not feeling well’, having a ‘common cold’ or feeling ‘a problem in (my) lungs’. Some reported seeking care specifically for a bad cough or prolonged coughing. One reported more intense symptoms “First it was cold, then there was difficulty breathing, fatigue, sweating, and insomnia,” [P08F_Gyangze]. Only one participant reported specifically seeking care with TB in mind, “I got sick. After there was blood in my sputum, I went to get checked up. I especially went in to get checked up for TB,” [P01M_Sangzhuzi].

Some participants reported feelings of indifference towards their diagnosis, with one saying “I didn’t have much feeling about this. Maybe I was coughing a bit at that moment, but I was not afraid,” [P02M_Sangzhuzi]. Another reported disbelief saying, “I didn’t believe it when the doctor told me…I still do not believe, but I still take the medications now,” [P04M_Sa’gya]. One patient reported worry and resolved to go through treatment saying “I was worried that I had TB last year. I thought that I must cure this disease without fear,” [P05M_Sangzhuzi]. Others worried about passing TB to their family members or children. One family treatment supporter reflected how her past experience informed her opinion of TB explaining, “I had TB four years ago and it healed. Therefore, I don’t think TB is a serious condition, the medication is good. The treatment is good,” [TS01_Gyangze]. However, one young woman reported that “I was a little nervous when diagnosed with TB…I feel that some people have prejudice towards me after I got this infectious disease,” [P18F_Tingri].

### Ability to seek: care pathways and options

Some participants reported first accessing a township hospital when experiencing mild symptoms. One such participant described how he was diagnosed at the township hospital but then “I did the sputum examination in the city hospital because the township hospital did not have this examination,” [P02M_Sangzhuzi]. Others described accessing multiple points of care, as one patient described first going to a township hospital which could only diagnose him with suspected TB, then a county hospital where he took “anti-infection treatment, got better, and went back home, thought I was healthy,” [P03M_Sa’gya]. During a subsequent visit from a mobile TB screening program which visited his village he was advised to visit the county hospital for an x-ray, computerized tomography (CT) scan and sputum check where he was then diagnosed with TB. Many had to travel as far away as Lhasa either to be diagnosed or to seek tertiary care, for example one participant described how,

“The TB diagnosis was first confirmed in the district hospital. After staying there for a period of time, I was discharged after gradually improving. I relapsed more than a month after being discharged from the hospital and went directly to Lhasa for treatment,” [P12M_Sangzhuzi]. Another reported that “people around me recommend me to go to the inland [to Lhasa] for treatment,” [P05M_Sangzhuzi].

Participants who reported first accessing care at a county hospital were immediately diagnosed with TB, either via x-ray and CT or via sputum test. One patient described how his diagnosis took multiple visits and a referral,

I went to the city hospital for a common cold treatment last year. I found I have TB condition from the CT examination, but the sputum test showed a negative result. The hospital recommended me not to take medicine. This year, when I get a common cold, the condition has become more serious again. Local hospital cannot confirm if that it is TB and the local hospital suggest me to go to [a provincial hospital in Lhasa] for an examination and I was diagnosed with the disease, [P05M_Sangzhuzi].

Another summed up his challenges seeking care at hospitals as, “Inconvenient transportation, long distance, insufficient knowledge and literacy so can't understand signage,” [P16M_Tingri].

Some participants first sought care using local complementary medicine known as Tibetan traditional medicine (TTM). Tibetan medicines are comprised of natural herbs found on the Tibetan plateau, and the practice is closely tied to local culture [33]. In Xizang, the health system includes both western and TTM hospitals and many patients routinely seek care at TTM hospitals for a variety of ailments [21]. A few participants with mild symptoms reported seeking TTM. One patient saw a Tibetan doctor and did not receive a diagnosis, however during an annual check-up offered by township hospitals (western medicine) he was referred to the county hospital and diagnosed with TB. One patient with severe symptoms sought care at a city TTM hospital and described how,

At the (city hospital), I did a chest x-ray and then I came here (County CDC). At that time, the water was frozen here and there was no way to do a sputum test, and so I went to the city hospital for the sputum test, [P01M_Sangzhuzi].

While there is formalized TTM in Xigaze, private TTM doctors are often consulted for symptom relief. Indeed, participants who reported seeking care in the western medicine system also reported taking TTM when first experiencing symptoms. One described how “I tried Tibetan medicine when I first started (having symptoms) … they weren’t very effective, the herbal medicine was mainly for subsiding cough.” [P08F_Gyangze]. Another reported taking Tibetan medicine after being diagnosed with TB explaining “Tibetan medicine was boiled in boiling water, the cough (got) slightly better,” [P04M_Sa’gya].

### Ability to reach: physical barriers and facilitators to accessing health services

Some participants described how their condition made them weak which made walking to access health services difficult, as one participant described “I am not very mobile, and I have to take a taxi in the city area,” [P09F_Sa’gya].

Participants also described limited transport options. Many participants described difficulties reaching the city hospital in Xigaze from rural areas, with journeys taking up to two days, which required renting a car or a paid carpool spot. Participants from Sangzhuzi, an urban district, reported fewer transport difficulties in reaching care, including shorter travel time and more transport options. While most patients reported a short or acceptable wait time for services, some patients reported lengthy waits. One participant from Sangzhuzi described,

I usually arrive at the hospital at 7:00 am, and I leave at 6:00 pm. I went to the queue (in the hospital) in the morning, and then there is another queue in front of the doctor’s office. Sometimes, I couldn't finish my examination in the radiology department because they are already off work when I get there… So, I need to go there the next day. Sometimes I even have to go to Lhasa. The total delay time is about one month, [P05M_Sangzhuzi].

Participants described ways in which they overcame the physical barriers to reaching care. Beyond paying for cars or taxis, this involved social support to get them to the care, with most who had transport challenges asking family members to transport them to the hospital. One participant was brought to the hospital for a check-up by the village doctor.

Participants described home visits as a facilitator that enabled them to reach health services and ongoing care. One participant described how “the village doctor came, on average once per month, one phone call per week, reminding them that they have responsibilities as well, that they can’t stop taking medication,” [P03M_Sa’gya].

### Ability to pay: subsidies and out of pocket payments

No participants reported catastrophic medical expenses, likely due to government programs providing free TB medications. One participant reported “The drugs are free. If I am hospitalized due to TB, the government covers most of the fees, and I think this is great,” [P02M_Sangzhuzi]. Another reflected how “The present treatment is good because we do not need to pay. If payments are required for the TB treatments, then there will be delays in TB treatments. We are relatively poor here,” [TS01_Gyangze]. One patient reported receiving subsidies to support nutrition, commenting “The disease control center sent out eggs and milk, where can be claimed four different times,” and reasoned that “All are distributed as the actual things because if money was distributed, people would use it to buy alcohol to drink and tobacco to smoke,” [P01M_Sangzhuzi].

However, despite these financial and material subsidies others described needing to pay for care. One participant described how although his medical cost was covered by the household account in the rural health insurance scheme, the account was shared between family members. He reported,

If there is money in the card (account) I do not have to pay… I take twenty out of my pocket, the government subsidize one hundred…depends on how many family members go see doctor, if not a lot of family members go see doctor it is enough, [P03_Sa’gya].

While medication is provided free of charge, participants largely reported out-of-pocket payment for check-ups and testing supplies such as sputum boxes, “phlegm checks,” blood tests and CT scans. No patients reported directly that these were unaffordable costs, and the majority of participants reported paying for expenses themselves. For some these costs were difficult, as one treatment supporter summarized, “The cost of blood test and ultrasound is more difficult for the family because there are four children in the family all go to school [so] the financial pressure is a little heavy.” [TS05_ Sa’gya]. Others described how testing costs necessitated choosing which tests to get, for example one participant explained, “Yes, I was told to do everything (x-ray and sputum test) in the beginning, but I think it is very burdensome economically so I only did chest radiograph,” [P06M_Sa’gya].

Other out of pocket costs related to care included the cost of TTM and transport. One participant described overnight travel expenses needed to confirm a TB diagnosis so that they could get their medications covered by the government,

The travel journey is not very convenient. I stayed in a hotel during the investigation at Xigaze, it is very inconvenient to get to the county hospital, to get to Xigaze is even farther. TB is a special outpatient clinic, requiring a diagnosis certificate at the municipal level or above; if there isn’t a diagnosis certificate, there is no way to reimburse the expenses, [P09F_Sa’gya].

One participant described the opportunity cost and lost income from travelling for medical care. He explained how “The difficulties are mainly time and the commute. Because if I go to the hospital, nobody would look after my crops,” [P06M_Sa’gya].

### Ability to engage

The majority of participants reported being satisfied with their experiences with their doctors and the information they provide. One participant described how,

I feel that (the doctors) are very authoritative, I would not doubt them, I would do whatever the doctors say… the doctors said, TB can be cured with medication if taken on time, so I feel assured, [P06M_Sa’gya].

However, another participant exemplifies how this type of authoritative relationship may inhibit open conversation. The participant explained,

P: I wanted to ask if I got this disease because of smoking, but I didn’t ask the doctor at that time.

I: Why didn’t you ask?

P: I was embarrassed to ask, [P01M_Sangzhuzi].

One family treatment supporter reported that they believed that village doctors provided better care because they understand the patient’s needs and lifestyle better, thus facilitating more open communication.

No participants reported receiving information on TB from other sources, however, one participant who is a family treatment supporter reported how her lived experience made her confident in the treatment her family member was receiving. She commented, “Because I had TB before, therefore I know about TB,” [TS01_Gyangze].

Successful TB treatment requires an understanding of the need for ongoing medication adherence to cure the infection and prevent drug resistance. All participants reported that their doctors informed them of the importance of adherence. As one participant reported, “The doctor told me not to stop taking the medicine, and TB can be cured with good medication adherence,” [P07M_Sangzhuzi]. However, fewer participants reported being told about potential side effects. One participant directly explained, “I only know about the dosage of intake and time of intake, I’m not aware of anything other than that,” [P08F_Gyangze].

The majority of participants reported that their doctors also provided them with lifestyle advice such as eating nutritious food, quitting smoking and avoiding alcohol. Participants reported being advised to drink boiled milk, avoid oily or fatty foods that would cause “yang excess,” as one participant described “The doctor said meat and spicy foods should be cautioned,” [P04M_Sa’gya]. After the start of the COVID-19 epidemic in China, most patients reported that COVID-19 response measures had no impact on their care seeking or treatment, yet some patients reported that their doctor advised them to not “spit phlegm out in public [and to] wear a mask,” [P14F_Gyangze] as part of broader public health measures. It is important to note, however, that in total Xizang only had one reported case of COVID-19 given stringent internal border control policies that characterized China’s national COVID-19 response.

### Ability to maintain care

Two patients reported serious side effects which required intervention from their doctor. One participant reported asking the County CDC to change medication after experiencing itchiness. Another described having to discontinue treatment,

I didn’t really feel anything, just that I had diarrhea after taking the medication, I couldn’t eat any meals and was very irritable…I did according to what the doctor said, no missed doses; after these side effects occurred, the doctor said to first stop the medication, [P09f_Sa’gya].

Some participants reported minor side effects including dizziness, nausea and dry eyes. However, these side effects caused one participant to reflect on their ability to maintain their course of treatment, “Recently, I feel a bit disgusting after taking medicine. Sometimes I think about whether I can cure TB in six months,” [P05M_Sangzhuzi]. Another described how “I worried about whether it will get better after taking the medication, but now [I’m] same as before,” [P17F_Tingri]. Overall patients reported being satisfied with their treatment and reported improving symptoms, which helped them continue treatment. One patient explained her reasoning as, “the treatment is very effective, my conditions have improved a lot,” [P08F_Gyangze].

The majority of patients described how social support from their families helped them to maintain their care. Families were described as providing financial support, transportation, providing and paying for mobile phones and supporting participants in using mobile phones to remain in contact with care providers. Families were also reported as key to ensuring participants took their medications. As one participant described, “Sometimes my family will remind me, not to forget taking the medication. Those who live with me all remind me,” [P03M_Sa’gya].

## Discussion

This qualitative study explored barriers and enablers to accessing and maintaining contact with TB care services in rural communities in Xigaze Prefecture, Xizang, China from the perspective of persons impacted by TB and their family members who support them. We highlight key contextual factors that may result in diagnostic delay and inability to maintain treatment (Table 3), for information on these barriers and enablers with illustrative quotes please see Additional file 1: Table S1. Using the results of this study, we synthesized an additional element to the access to care framework salient to access to TB care. ‘Ability to maintain care’ is an important construct in light of long treatment courses and side effects which may limit abilities to access ongoing care and in turn impact on the uptake of interventions to improve quality of TB care.

**Table 3:** Contextual barriers and enablers to tuberculosis care in Xigaze, Xizang

Domain	Barrier	Enabler
Ability to perceive	• Indifferent attitude towards TB • Disbelief about diagnosis	• Knowledge of TB • Past experience with TB
Ability to seek	• Multiple referrals • Seeking only symptom relief from private TTM doctors	• Integrated public TTM and western care
Ability to reach	• Symptoms of TB • Limited transport • Wait times for services • Literacy barriers	• Family support to help with transport • Village doctors • Home visits
Ability to pay	• Shared health insurance scheme amongst family members • Out-of-pocket payment for check-ups, testing supplies (sputum boxes), blood tests and CT scans • Transportation costs including hotel stays • Income lost from travelling for medical care	• Free medicines • Hospitalization coverage • Nutrition subsidies • Financial support from family
Ability to engage	• Authoritative patient-provider relationship • Less information provided on potential medication side-effects	• Authoritative patient-provider relationship • Village doctors • Lifestyle advice
Ability to maintain care	• Side effects from medications • Worry about whether medications will be effective	• Improvement in symptoms • Support from family members reminding them to take their medications and connect with healthcare providers

*CT* computerized tomography; *TB* tuberculosis; *TTM* Traditional Tibetan Medicine

### Increasing culturally tailored care

Only one participant described purposefully seeking care because he believed he had symptoms of TB. Given the high rates of TB in Xigaze, there is a need for ongoing culturally tailored and linguistically compatible TB education to address gaps in awareness, reduce diagnostic delay, and improve the quality of TB care. These campaigns must also consider the role of TTM in communities. As many people seek TTM as primary care, public health efforts should involve the TTM system to reduce diagnostic delay [21]. Others have reported similar patient experiences in the region and emphasized the need for links between TTM and western TB care [26]. Similar experiences of first seeking traditional healers or herbal remedies for TB symptoms have been reported globally including in Africa, South Asia and South America [34–38]. Others have reflected on how gender, income level or occupation, as well as health system factors may drive people to first seek  complementary or traditional medicine in LMICs [39, 40]. In China, and globally, a lack of awareness of TB and visiting traditional medicine providers has been linked to diagnostic delay [41–43]. Improving the quality of TB care requires more stronger linkages between traditional medicine, informal providers, and the formalized medical system to ensure patients can access and maintain appropriate TB treatment [44, 45].

### Minimizing complex pathways to care

Participants also described complex pathways to care, with most patients needing to visit two hospitals to receive a diagnosis. Another study in China found that persons living in rural communities and those seeking care for TB at lower level clinics or hospitals had greater diagnostic delay [46]. Studies from rural areas globally, including other high burden settings such as India, Indonesia, Nigeria, and the Philippines have shown similar patterns of rural diagnostic delay and complex pathways to treatment initiation [47–50]. Other studies from China have shown that the integrated TB care model where a county/district general hospital provides TB care, as in our setting, is associated with less diagnostic delay compared to the traditional model where the CDC provides TB care [51]. Once diagnosed, patients in our study reported numerous factors which may result in loss-to-follow-up and inability to maintain treatment such as transport, mobility, cost, side effects or lack of social support. These have been identified in other studies as factors associated with non-adherence to treatment in China [52–54]. Our findings and this complementary evidence further emphasize the need for TB programs, and health systems overall, to minimize complexity in TB testing and treatment pathways, while embracing and taking steps to address the complexity of the socioeconomic needs that persons receiving treatment must have met to ensure initiation and continuity of care.

### Reducing out of pocket payments

Subsidized treatment for TB is a key facilitator to accessing care in Xigaze. However, patients reported needing to pay out of pocket for tests and other aspects of care. Previous studies in China have identified high out-of-pocket expenditures for TB patients under the rural health insurance schemes, highlighting the need for continued action towards comprehensive universal health coverage (UHC) as a way to ensure access and continuity of care [55–57]. Indeed, there is ample global evidence from South Asia, South East Asia, and sub-Saharan Africa regarding how out-of-pocket expenditures limit TB diagnosis, treatment initiation, and maintenance and contribute to the catastrophic cost of TB care [58–60]. To ensure patients are able to afford all aspects of their care, it is important to consider the non-trivial costs associated with accessing care in rural and remote settings. Other qualitative studies in rural China have similarly reported that transport costs and travel distance influenced diagnostic delay and retention in care [61]. Others have described how a pilot program in China for such a subsidy enabled rural TB patients to complete required visits [62]. Persons impacted by TB can be further supported to maintain treatment when provided holistic support that addresses the many incurred costs in reaching healthcare facilities and maintaining health and well-being during TB treatment in the community [63].

### Promoting social and family support in care

Underpinning access and maintenance of TB care is social support which facilitated participants ability to reach, ability to pay and ability to maintain care. In Xizang, large families live together in multi-generational households and all participants discussed their families as providing social support in relation to their care. Other studies in Xizang, as well as other areas of China, have highlighted the importance of social and family support in TB care on adherence, particularly amongst newly diagnosed patients [26, 64]. A 2014 review reported that family treatment observation combined with health worker home visits, as in our setting, can achieve high cure rates if overall treatment is closely monitored and well-connected to the healthcare system [65]. While our findings underscore how families are a source of social support enabling treatment and continuity of care, they also emphasize global findings that social and financial costs are collectively borne by households [66, 67]. Thus, interventions to mitigate costs incurred in accessing or maintaining care can reap benefits at the household level.

### Strengths and limitations

A strength of this manuscript is our exploration of rural and remote participants’ perspectives on their abilities to access and engage with TB care. This exploration of factors is strengthened by our adaptation of Levesque’s patient-centered access-to-care framework. This study contributes to the limited body of knowledge on perspectives of persons affected by TB in Xizang and a particular strength is our use of semi-structured interviews in Tibetan language. This allowed for the exploration of patient perceptions and care seeking in a more approachable and culturally sensitive way.

An important limitation of the study is desirability bias whereby participants present a more positively framed recounting of their experiences. We attempted to mitigate this by reassuring participants of the confidentiality of their responses. Such bias likely had minimal impact on our study as participants were candid in sharing their challenges. Also, while we strived to recruit participants from different regions and with a diversity of experience, by nature of our recruitment strategy, our sample of participants is likely to have over-selected those who are already receiving health services. Thus, we may be missing the perceptions of those who are least connected to the health system.

## Conclusions

This qualitative study has identified enablers and barriers to accessing TB related services in rural communities in Xigaze Prefecture, Xizang, China from the perspective of persons impacted by TB. Initial and ongoing access to TB care in Xigaze could be strengthened by ongoing culturally tailored educational campaigns to increase awareness, partnerships with TTM providers, providing comprehensive treatment subsidies and strengthening the role of family members in comprehensive TB care.

## Supplementary Information

**Additional file 1: Table S1.** Selected supporting quotes on contextual barriers and enablers to TB care in Shigatse, Tibet.

## Data Availability

The anonymized data that support the findings of this study are available on request from the corresponding author XW. The data are not publicly available due to them containing information that could compromise research participant privacy/consent.

## Abbreviations

CDC: Centres for Disease Control
CT: Computerized tomography
DOT: Directly Observed Treatment
LMICs: Low-and-middle-income countries
NTP: National Tuberculosis Program
TTM: Traditional Tibetan medicine
TB: Tuberculosis
UHC: Universal Health Coverage
