# Supplementary material for: A qualitative study of perspectives on access to tuberculosis health services in Xigaze, China
Source: Infect Dis Poverty. 2021 Sep 20;10:120. doi: 10.1186/s40249-021-00906-4 (PMC8451167; doi:10.1186/s40249-021-00906-4)
Supplement: Supplementary file 1 — Additional file 1: Table S1. Selected supporting quotes on contextual barriers and enablers to TB care in Shigatse, Tibet. [file 40249_2021_906_MOESM1_ESM.docx]

Supplementary File 1. Selected supporting quotes on contextual barriers and enablers to TB care in Shigatse, Tibet.

| **Domain** | **Barrier** | **Enabler** |
| --- | --- | --- |
| **Ability to perceive** | - Indifferent attitude towards TB - Disbelief about diagnosis | - Knowledge of TB - Past experience with TB |
|  | “A: When you were diagnosed with TB? What was your thought at that moment and what was your feeling?  B: I didn’t have much feeling about this. Maybe I was coughing a bit at that moment, but I was not afraid.” [P02M_Samzhubze]  “A: When the doctor told you it was tuberculosis, did you have any thoughts?  B: I didn’t believe it when the doctor told me, but before my relatives had symptoms of tuberculosis. I still do not believe, but I still take the medications now.” [P04M_Sa’gya] | “I know the disease quite well. The doctor at Tibetan Medicine Hospital also told me previously, the teacher there said I should go to the immunization station for more information on tuberculosis, I went and inquired.” [P06M_Sa’gya]  “They [the patient] thought that if diagnosed of tuberculosis, there would be no treatment and will die. Now, they are slowly taking tuberculosis medications and are slowly getting better.” [TS02M_Tingri]  “I had TB four years ago and it healed. Therefore, I don’t think TB is a serious condition. The medication is good. Treatment is good.” [TS01_Gyantse] |
| **Ability to seek** | - Multiple referrals - Seeking only symptom relief from private TTM doctors | - Integrated public TTM and western care |
|  | “I couldn’t get a diagnosis at the township hospital, I was recommended to get a check-up in bigger hospitals, maybe it was tuberculosis, then the doctor on round-trip visits took the x-ray, and suspected me of past contraction with tuberculosis, referred me to get checked up at bigger hospital, which then was I officially diagnosed.” [P03M_Sa’gya]  “The tuberculosis diagnosis was first confirmed in the district hospital. After staying there for a period of time, I was discharged after gradually improving. I relapsed more than a month after being discharged from the hospital and went directly to Lhasa for treatment. In Lhasa, I went to the People’s Hospital of the Autonomous Region first, then transferred to the Third People’s Hospital for treatment, and started taking medicine after returning home.” [P12M_Samzhubze]  “I saw a Tibetan doctor and there was no diagnosis. I was diagnosed with tuberculosis during my 18-year farmer’s health check-up. After, the final diagnosis was made when I went to the county people’s hospital to accompany my wife to see the doctor.” [P04M_Sa’gya]  “At first, I went to the county people’s hospital, the county people’s hospital asked us to go the Shitgatse people’s hospital for the examination… Also the county people’s hospital, but didn’t go to any others.” [P09F_Sa’gya]  “ A: Have you ever tried Traditional Tibetan Medicine or other folk remedies?  B: I tried Tibetan medicine when I first started [having the symptoms], nothing else other than that.  A: Was the Tibetan medicine effective?  B: They weren’t very effective, the herbal medicine was mainly for subsiding coughs.” [P08F_Gyantse] | “I went to Shigatse People’s Hospital, Dingri County Hospital, and Shigatse Tibetan Hospital. After they found out, because they said to go to the big hospital for a check, I went to the Shigatse People’s Hospital for an examination. The doctor said there was a little tuberculosis, and then asked I went to the Tibetan hospital to draw blood. I have done blood sampling, CT, X-ray, and sputum examination. The final diagnosis was confirmed at Shigatse Tibetan Hospital.” [P10F_Tingri]  “A: Has the patient taken Tibetan medicine or any other remedies? B: In the beginning, patient has taken Tibetan medicine. A： Did it show results? B: No.” [TS01_Gyantse]  “ A: Have you ever tried Traditional Tibetan Medicine or other folk remedies?  B: I tried Tibetan medicine when I first started [having the symptoms], nothing else other than that.  A: Was the Tibetan medicine effective?  B: They weren’t very effective, the herbal medicine was mainly for subsiding coughs.” [P08F_Gyantse]  “A: Before, how much money was spent on taking Tibetan medications?  B: I took it for 3 years, around 10,000 in total. To treat the old cough. After the diagnosis of tuberculosis, around 2000 was spent on taking Tibetan medicine, all self paid.  A: Do you think you can afford Tibetan medicine taken before?  B: It’s okay.” [P04M_Sa’gya] |
| **Ability to reach** | - Symptoms of TB - Limited transport - Wait times for services - Literacy barriers | - Family support to help with transport - Village doctors - Home visits |
|  | “[I go to] township hospital, I don't have much strength when walking, legs hurt, difficulty breathing, fatigue, wanting to rest constantly.” [P01M_Samzhubze]  “The transportation is not very convenient. I don’t know how to drive electric vehicles and need other people to give a ride.” [P04M_Sa’gya]  “The travel journey is not very convenient. I stayed in a hotel during the investigation at Shigatse, it is very inconvenient to get to the county hospital, to get to Shigatse is even farther. Lung tuberculosis is a special outpatient clinic, requiring a diagnosis certificate at the municipal level or above; it there isn’t a diagnosis certificate, there is no way to reimburse the expenses.” [P09F_Sa’gya]  “I need to be in the queue for a day to see a doctor. I went to the queue (in the hospital) in the morning, and then there is another queue in front of the doctor’s office. Sometimes, I couldn't finish my examination in the radiology department because they are already off work when I get there (completing other examinations). My examinations for this time was delay for a month. And I couldn’t catch up for many examinations. So, I need to go there the next day. Sometimes I even have to go to Lhasa. The total delay time is about one month.” [P05M_Samzhubze].  The participant listed their difficulties including “Inconvenient transportation, long distance, insufficient knowledge and cultural level [at the hospital[, can't understand the signs.” [P16M_Tingri]. | “My son owns a car, so he usually drives me to the hospital. When he is not free, I will go to the hospital by bus…The doctor in my village will come to my house and visit me every week. The doctor in our town will contact me on the phone.” [P02M_Samzhubze]  “A: Did the village doctor communicate with you? How often did he contact you?  B: Yes, once every week. He would call to ask if I took the medication or not.” [P01M_Samzhubze]  “The village doctor called, the township hospital doctor did not call, when they are busy normally, [I] would call the village doctor first, and the village doctor would subsequently call the township hospital doctor.” [P03_Sa’gya]  “A: Do you know the precautions of the TB drug that you are taking?  B: Yes.  A: Who told you these precautions?  B: The village doctor told me.  A: Did that village doctor provide any advice based on these precautions?  B: The village doctor told me to take medicine on time, improve my diet and quit smoking.” [P05M_Samzhubze]  “The village doctor created a group chat, if there is any problem I can just talk in the group chat, it is rather convenient.” [P06M_Sa’gya]  “The village doctor is better [because they] understand the patient’s conditions.” [TS03F_Tingri] |
| **Ability to pay** | - Shared health insurance scheme amongst family members - Out-of-pocket payment for check-ups, testing supplies (sputum boxes), blood tests and CT scans - Transportation costs including hotel stays - Income lost from travelling for medical care | - Free medicines - Hospitalization coverage - Nutrition subsidies - Financial support from family |
|  | “A: What about the cost for treating your tuberculosis?  B: I can get reimbursed, health card, if there is money in the card I do not have to pay.  A: How much money is in the card?  B: I take twenty out of my pocket, the government subsidizes one hundred.  A: So only one hundred and twenty RMB in the card, is it enough?  B: Depends on how many family members go see doctor, if not a lot of family member go see doctors it is enough.” [P03_Sa’gya]  “A: So you were ordered to do liver function check, chest radiograph and phlegm check at the start of the diagnostic process, but you didn’t do phlegm check after?  B: Yes, I was told to do everything in the beginning, but I think it is very burdensome economically so I only did chest radiograph.” [P06M_Sa’gya]  “A: The district hospital now has a sputum test room. How much does a sputum box cost? B: In the past, the sputum box was free. Now I don't know if the hospital sputum box will charge.  A: What else do you spend on treating TB?  B: In addition to a sputum box of two yuan, I need to use 3 sputum box per test so a total of six yuan, there is a 4 Yuan registration fee, and 15 yuan for sputum check….” [P07M_Samzhubze]  “A: The drugs provided in the CDC are free of charge. Are you paying for the cost of going to Lhasa? B: Yes, I pay for the cost.” [P05_Samzhubze]  “The difficulties are mainly time and the commute. Because if I go to the hospital, nobody would look after my crops… Sometimes I would have to order the whole car, and it costs about 300 [Yuan]. If there are carpool headed the same destination then it would be only 50 something, if there isn’t, then I would have to order one… Basically, there’s always line up at the hospital, if I line up early in the morning it is ok, takes about the whole morning.” [P06M_Sa’gya] | “The drugs are free. If I am hospitalized due to TB, the government covers most of the fees, and I think this is great.” [P02M_Samzhubze]  “The present treatment is good because we do not need to pay. If payments are required for the TB treatments, then there will be delays in TB treatments. We are relatively poor here.” [TS01F_Gyantse]  “B: The medications are free, but you get charged for check up. The new medication would cost you, but you get reimbursed after. A: How much have you spent in total relating to treatment? B: I was charged about 7000 RMB on hospitalization, but I only paid for 1000 RMB, the rest is reimbursable.” [P08F_Gyantse]  “Tuberculosis has 400 yuan in nutrition fees. The disease control center sent out eggs and milk, where can be claimed 4 different times. All are distributed in as the actual things because if money was distributed, people would use it to buy alcohol to drink and tobacco to smoke.” [P01M_Samzhubze]  “A: How much it cost for those TB examinations such as a CT scan?  B: Around 500 RMB. The examinations include CT scan and sputum examination  A: Who pays for the medical bills?  B: My family, mostly my son.” [P02M_Samzhubze]  “[Patient described their out of pocket costs]  A: Are you paying for these costs or your family?  B: My family help me to pay.” [P07M_Samzhubze] |
| **Ability to engage** | - Authoritative patient-provider relationship - Less information provided on potential medication side-effects | - Authoritative patient-provider relationship - Village doctors - Lifestyle advice |
|  | “I feel that [the doctors’] are very authoritative, I would not doubt them, I would do whatever the doctors say… the doctors said, tuberculosis can be cured with medication if taken on time, so I feel assured,” [P06M_Sa’gya].  “A: Do you have any questions about your condition? Have you ever asked the doctor about the condition?  B: No questions, I wanted to ask if I got this disease because of smoking, but I didn’t ask the doctor at that time.  A: Why didn’t you ask?  B: I was embarrassed to ask.” [P01M_Samzhubze]  A patient described how he previously had visited a doctor who told him to stop taking his medication, it was unclear whether this was a professional or informal provider, the patient described “O just wondered if the doctor couldn't understand and I doubted the doctor's ability. But I know the doctor. He told me that I didn't need to take medicine…He said that I didn't need to take medication, so I followed…he told me there is no need to take medicine after he saw the CT picture…Once I get to the county CDC, I feel that I need to treat this TB symptom seriously. Also, I have a good impression of those doctors.” [P05M_Samzhubze] | “[The patient] feared that it will not be cured. After listening to the doctors from the county hospital and actively cooperating with treatment, the patient was not so afraid.” [TS04F_Samzhubze]  “B: The doctor said to not smoke, to not eat oily foods, to not drink alcohol.” [P01M_Samzhubze]  “The doctor also said I have to quit smoking, I told him that I can not quit completely, I can only smoke less,” [P06M_Sa’gya].  “The doctors told me eat a lot of nutritious food, and keep away from food that will cause Yang excess, such as lamb, I’ve followed through with the doctor’s advice,”  “Satisfied, the doctor often reminds me to take medications on time, answers my questions and worries, and provided some health education to support the my mental health.” [P18F_Tingri]  “The doctor is very good. He has some suggestions on diet and medication, and understands my concerns…Teacher Deji (Tingri disease control teacher) and village doctor. They recommended diets, sputum management, and to wear a mask during interpersonal communication. And to follow these strictly.” [P10_Tingri] |
| **Ability to maintain care** | - Side effects from medications - Worry about whether medications will be effective | - Improvement in symptoms - Support from family members reminding them to take their medications and connect with healthcare providers |
|  | “I find a bit difficult to swallow the pills,” [P02M_Samzhubze].  “Not really any problems. It’s just a little nervous to take, dizziness, nausea, dry eyes,” [P04M_Sa’gya].  ‘Recently, I feel a bit disgusting after taking medicine. Sometimes I think about whether I can cure TB in 6 months,” [P05M_Samzhubze]  “Combinatory medication, when I take this medication, it would itch, that medication, it wouldn't itch if you take three tablets daily. I told the County CDC myself; the County CDC gave me new medication, it didn’t itch,” [P03M_Sa’gya].  “I didn’t really feel anything, just that I had diarrhea after taking the medication, I couldn’t eat any meals and was very irritable…I did according to what the doctor said, no missed doses; after these side effects occurred, the doctor said to first stop the medication,” [P09f_Sa’gya] | “I felt fatigued, I was coughing, my conditions improved later on.” [P15M_Gyantse]  “When I started taking tuberculosis drugs, I felt a little in a daze; now I have adapted to it without any side effects.” [P12M_Samzhubze]  “I felt nervous at the time of diagnosis, weak, sleepy. Afterwards, I became used to it.” [P11F_Tingri]  “After diagnosed with tuberculosis, I was a little nervous; also had some physical impact. After anti-tuberculosis treatment, my body has improved significantly.” [P17F_Tingri]  “After taking medications, their [the patient’s] condition gradually improved. In the past, their movement was inconvenient, life was affected by it…they were nervous in the beginning, but improved after taking medicine and gradually improved in terms of diet.” [TS05F_Sa’gya]  “Family reminds. Mainly daughter-in-law, reminds me every morning.” [P16M_Tingri]. |
